# Quantitative Magnetic Resonance Imaging for Neurodevelopmental Outcome Prediction in Neonates Born Extremely Premature—An Exploratory Study

**DOI:** 10.1007/s00062-023-01378-9

**Published:** 2024-01-30

**Authors:** Victor U. Schmidbauer, Mehmet S. Yildirim, Gregor O. Dovjak, Katharina Goeral, Julia Buchmayer, Michael Weber, Patric Kienast, Mariana C. Diogo, Florian Prayer, Marlene Stuempflen, Jakob Kittinger, Jakob Malik, Nikolaus M. Nowak, Katrin Klebermass-Schrehof, Renate Fuiko, Angelika Berger, Daniela Prayer, Gregor Kasprian, Vito Giordano

**Affiliations:** 1 Department of Biomedical Imaging and Image-guided Therapy, Medical University of Vienna, Waehringer Guertel 18–20, 1090 Vienna, Austria; 2 Comprehensive Center for Pediatrics, Department of Pediatrics and Adolescent Medicine, Division of Neonatology, Pediatric Intensive Care and Neuropediatrics, Medical University of Vienna, Waehringer Guertel 18–20, 1090 Vienna, Austria; 3 Department of Neuroradiology, Hospital Garcia de Orta, Av. Torrado da Silva, 2805-267 Almada, Portugal

**Keywords:** Brain Stem, Extremely Premature, Magnetic Resonance Imaging, Newborn, Prognosis

## Abstract

**Purpose:**

Neonates born at < 28 weeks of gestation are at risk for neurodevelopmental delay. The aim of this study was to identify quantitative MR-based metrics for the prediction of neurodevelopmental outcomes in extremely preterm neonates.

**Methods:**

T1-/T2-relaxation times (T1R/T2R), ADC, and fractional anisotropy (FA) of the left/right posterior limb of the internal capsule (PLIC) and the brainstem were determined at term-equivalent ages in a sample of extremely preterm infants (*n* = 33). Scores for cognitive, language, and motor outcomes were collected at one year corrected-age. Pearson’s correlation analyses detected relationships between quantitative measures and outcome data. Stepwise regression procedures identified imaging metrics to estimate neurodevelopmental outcomes.

**Results:**

Cognitive outcomes correlated significantly with T2R (*r* = 0.412; *p* = 0.017) and ADC (*r* = −0.401; *p* = 0.021) (medulla oblongata). Furthermore, there were significant correlations between motor outcomes and T1R (pontine tegmentum (*r* = 0.346; *p* = 0.049), midbrain (*r* = 0.415; *p* = 0.016), right PLIC (*r* = 0.513; *p* = 0.002), and left PLIC (*r* = 0.504; *p* = 0.003)); T2R (right PLIC (*r* = 0.405; *p* = 0.019)); ADC (medulla oblongata (*r* = −0.408; *p* = 0.018) and pontine tegmentum (*r* = −0.414; *p* = 0.017)); and FA (pontine tegmentum (*r* = −0.352; *p* = 0.045)).

T2R/ADC (medulla oblongata) (cognitive outcomes (R^2^ = 0.296; *p* = 0.037)) and T1R (right PLIC)/ADC (medulla oblongata) (motor outcomes (R^2^ = 0.405; *p* = 0.009)) revealed predictive potential for neurodevelopmental outcomes.

**Conclusion:**

There are relationships between relaxometry‑/DTI-based metrics determined by neuroimaging near term and neurodevelopmental outcomes collected at one year of age. Both modalities bear prognostic potential for the prediction of cognitive and motor outcomes. Thus, quantitative MRI at term-equivalent ages represents a promising approach with which to estimate neurologic development in extremely preterm infants.

**Supplementary Information:**

The online version of this article (10.1007/s00062-023-01378-9) contains supplementary material, which is available to authorized users.

## Introduction

Preterm births account for approximately 11% of all deliveries worldwide [1]. Prematurity is considered a risk factor for neurodevelopmental delay [2, 3]. There is a relationship between gestational age (GA) at birth and neurologic outcome, with children born at a lower GA bearing a higher risk for poor future cognitive/language abilities and impaired motor skills [2–4]. Thus, among subjects with a preterm birth history, neonates formerly born extremely premature (< 28 weeks of gestation (week’s completed gestation)) represent the most fragile patient population in terms of potentially adversely affected neurocognitive development [2–4]. While the number of extreme preterm-delivery survivors steadily increases, reliable, non-invasively determined biomarkers for neurologic outcome prediction are still scarce [4–7].

Routine MRI at term-equivalent ages in infants born at < 28 weeks of gestation has become an accepted diagnostic procedure [8]. While conventional MRI contrasts serve as a sensitive tool for the qualitative, prognostic evaluation of pathologies of the preterm brain, advanced acquisition strategies (i.e., relaxometry-based MRI and DTI) provide the opportunity to supply multi-parametric, quantitative data to the radiological assessment [9–13]. Several studies suggest a relationship between the evolution of quantitative, relaxation time properties and DTI parameters throughout development and the biochemical processes (e.g., myelination) of brain maturation [10, 11, 14, 15]. However, there is still a lack of information about the prognostic value of these metrics with regard to neurologic outcome in infants after preterm birth.

The aim of this exploratory study was to investigate the potential value of relaxation time metrics (i.e., T1-/T2-relaxation times (T1R/T2R)) and DTI parameters (ADC/fractional anisotropy (FA)) for neurologic outcome prediction in a cohort of extremely preterm infants. For that purpose, quantitative metrics were determined in early-myelinating neonatal brain regions at term-equivalent ages. Correlational analyses were applied to detect relationships between quantitative MR data and cognitive, language, and motor outcomes collected at one year corrected-age. Furthermore, stepwise regression procedures were conducted to identify the imaging metrics most predictive for neurodevelopmental outcomes.

## Materials and Methods

### Ethical Approval

The local institutional review board approved the protocol of this retrospective study. All guardians provided written, informed consent for neonatal MRI before scanning.

### Study Cohort

At our institution, brain MRI is performed routinely at approximately term-equivalent ages in infants born < 28 + 0 (i.e., ≤ 27 + 6 weeks + days) of gestation [2]. Between June 2017 and September 2020, 41 extremely preterm neonates (without structural cerebral pathologies) were examined at term-equivalent ages. Neonatal neuroimaging was performed at the Department of Neuroradiology of a tertiary care medical center. All infants were referred for brain MRI by the Department of Pediatrics and Adolescent Medicine of the same tertiary care hospital. Only neonates in whom MRI was perceived to be “without pathological brain findings” (excludes minor delays in brain maturation (i.e. opercularization, etc.) (in consideration of extreme prematurity)), were enrolled in this investigation. Table 1 provides detailed information about the subjects included.

**Table 1:** Characteristics of included infants

	Extremely Preterm Neonates^a, b^ (*n* = 33)
*Perinatal/Postnatal Characteristics*	
Female	*n* = 16
Male	*n* = 17
GA at Birth (weeks + days)^c^	26 + 1, *SD* = 0 + 3
Vaginal Delivery	*n* = 3
Caesarean Delivery	*n* = 30
Birth Weight (gram)^c^	833, *SD* = 242
Singleton Pregnancy	*n* = 21
Multiple Pregnancy	*n* = 12
Surfactant Received	*n* = 33
Retinopathy of Prematurity	*n* = 15
Bronchopulmonary Dysplasia	*n* = 6
Necrotizing Enterocolitis	*n* = 2
Patent Ductus Arteriosus	*n* = 4
Perinatal Asphyxia^d^	*n* = 8
PMA at MRI (weeks + days)^c^	37 + 2, *SD* = 1 + 3
*Neonatal Mortality Assessment*	
CRIB II Score^c^	11, *SD* = 3
*Maternal Characteristics*	
Age at Delivery (years)^c^	32, *SD* = 5
Preeclampsia	*n* = 7
Intra-Amniotic Infection (Chorioamnionitis)^e^	*n* = 4
Gestational Diabetes Mellitus	*n* = 1
*Assessment of Neurodevelopmental Outcomes at 12 Months Corrected-Age*	
Cognitive Outcome Scores^c, g^	94, *SD* = 16
Language Outcome Scores^c, g^	99, *SD* = 12
Motor Outcome Scores^c, g^	94, *SD* = 14

*CRIB* Clinical Risk Index for Babies, *GA* Gestational age, *MRI* Magnetic resonance imaging, *PMA* Post-menstrual age

^a^ Studied infants have been reported previously (MDME- and DTI-based data). However, these investigations focused on different research objectives^9,10,21^

^b^ Born at < 28 weeks of gestation

^c^ Data presented as mean accompanied by standard deviation (*SD*)

^d^ Based on Apgar scores, umbilical cord pH, first blood gas analysis (pH, base excess, and lactate), and clinical presentation (no evidence/absence of asphyxia-related brain injury)

^e^ Based on placental histology, bacterial culture, inflammatory markers, and clinical presentation

### Neonatal MRI, Quantitative Sequences, and Post-Processing

MR-based neuroimaging was performed according to the institutional feed-and-wrap protocol. To prevent movement-related artifacts, neonates were bedded on a vacuum mattress. All infants were examined using a standardized neonatal MR protocol (Supplementary Table 1) on one 1.5 Tesla MR system (Ingenia, Philips Healthcare). To obtain quantitative data, multi-dynamic multi-echo (MDME) sequences (axial plane) and DTI sequences (axial plane) (flip angle, 90°) were acquired (Table 2). Via two repeated acquisition phases (*phase I*: a slice-selective saturation pulse (flip angle, 120°) saturates one section; *phase II*: slice-selective refocusing pulses (flip angle, 180°) and excitation pulses (flip angle, 90°) evolve series of spin echoes for another section), the MDME sequence determined the tissue-specific T1R/T2R properties [10, 16–18]. MR data post-processing was performed using the IntelliSpace Portal (Version 10; Philips Healthcare) (DTI sequences) and SyMRI (Synthetic MR AB; Version 11.2) (MDME sequences).

**Table 2:** Quantitative sequences

	DTI	MDME
Acquisition Time (min)	05:26	05:24
Directional Resolution	32	–
*b *Value (s/mm^2^)	0/800	–
Slice Number	40	22
Gap (mm)	0	1
Sense Factor	2	2
Pixel/Hz	5.310/40.9	1.366/159.0
EPI Factor^a^/Echo Train^b^	33	10
Field-of-View (mm)	164 × 164 × 100	200 × 165 × 109
Acquired Voxel Size(mm)	2.41 × 2.48 × 2.50	0.89 × 1.04 × 4.00
Echo Time (ms)	88	13; 100
Repetition Time (ms)	2435	3309

*DTI* Diffusion-tensor imaging; *EPI* Echo-planar imaging; *MDME* Multi-dynamic multi-echo

^a^ Applies to diffusion-tensor imaging sequences

^b^ Applies to multi-dynamic multi-echo sequences

### Determination of Quantitative MR Data at Term-Equivalent Ages

**Fig. 1:**
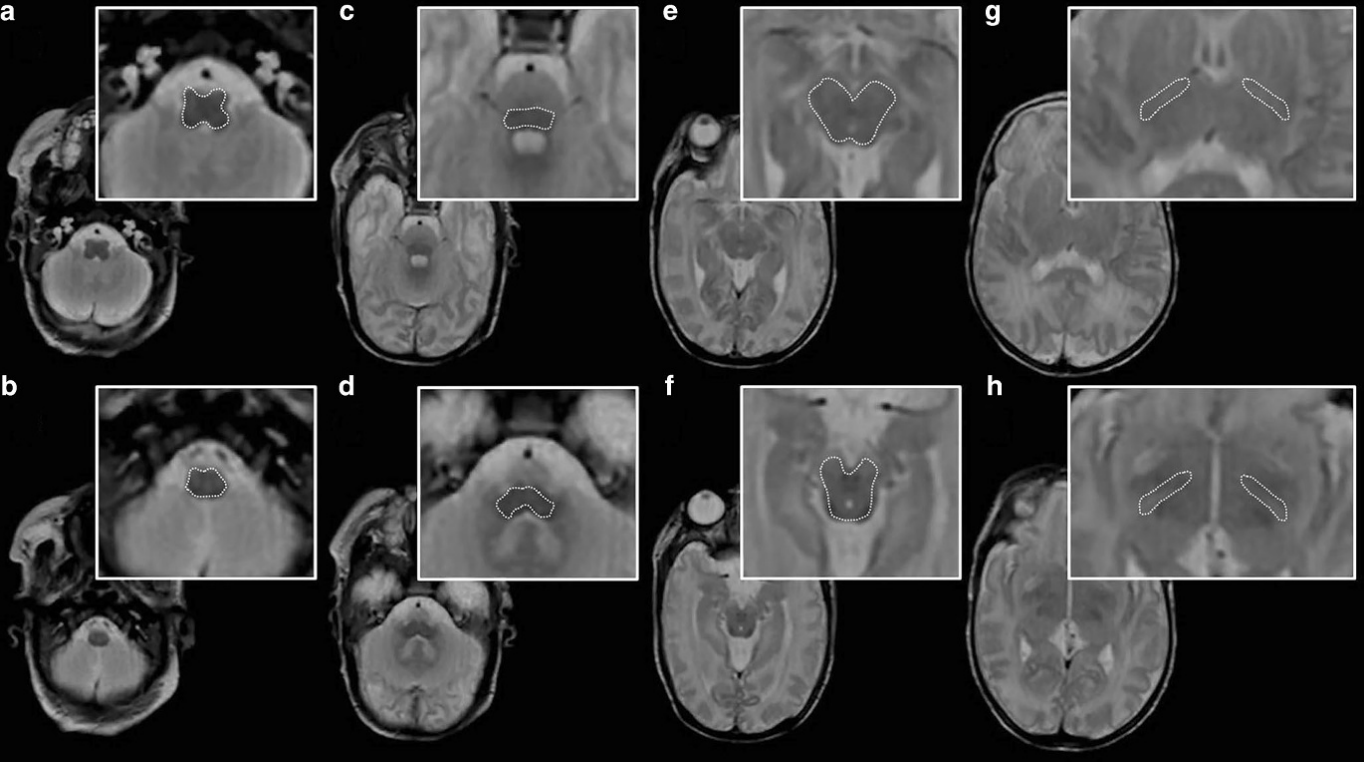
ROI drawings in a female infant (GA at birth: 27 + 5; MRI at 35 + 6) on a SyMRI-based, T2-weighted MR contrast (repetition time (TR)/echo time (TE): 4500/100 ms). Wherever applicable, two separate measurements were performed at different levels for each brain area of interest: e.g., medulla oblongata (**a** level of inferior olivary nucleus; **b** level of gracile and cuneate nucleus); pontine tegmentum (**c** level of superior olive; **d** level of the pontine vestibular nuclei); midbrain (**e** level of superior colliculi/intercollicular section; **f** level of inferior colliculi); and PLIC (**g** level of the interventricular foramen; **h** level of the third ventricle)

T1R (ms), T2R (ms), ADC (10^−3^ mm^2^/s), and FA parameters were determined in five neonatal brain regions of interest (ROI) that are considered to hold the highest quantities of myelin deposited at term-equivalent ages: the left posterior limb of the internal capsule (PLIC); the right PLIC; the midbrain; the pontine tegmentum; and the medulla oblongata [19–21]. Two different observers (rater 1, with four years of experience, and rater 2, with two years of experience with neonatal brain MRI) performed ROI placement manually on SyMRI-generated maps and DTI-based data. Wherever applicable, two separate ROIs were drawn at different levels for each brain area of interest (performed in a consensus reading). Alternatively—e.g., in case multiple sections were not available, or there was poor delineability of ROIs at a given level, etc.—two measurements were performed on one appropriate MR image slice. ROI placement is demonstrated in Fig. 1. The calculated mean values for DTI/relaxation time metrics based on both measures were used for further analyses.

### Assessment of Neurodevelopmental Outcome at One Year of Age

The neurodevelopment of former extremely preterm neonates was evaluated at 12 months corrected-age. One certified clinical psychologist with 30 years of experience performed outcome assessment. Infants were tested according to the *Bayley Scales of Infant and Toddler Development*, third edition (BSID-III), which is considered the gold standard for the assessment of cognitive, language, and motor outcomes in these patients (outcome scores > 85 are considered normal) [22, 23]. The corresponding scores for each developmental aspect were used for further analyses [23].

### Statistical Analyses

SPSS-based (SPSS Statistics for Macintosh (Version 27.0; IBM)) statistical analyses were performed as proposed by an expert in biomedical statistics, with 30 years of experience in the field. The significance level was set at α = 5% (*p* < 0.05).

An intra-class correlation coefficient (ICC) was calculated to provide an overview of the overall agreement of the quantitative metrics determined by both observers. ICC results were interpreted as described by Koo and Li (< 0.5: poor; 0.5 to 0.75: moderate; 0.75 to 0.9: good; and > 0.9: excellent) [24].

Pearson’s correlation analyses were performed to identify significant relationships between the quantitative measures determined at term-equivalent ages and the outcome scores for cognitive, language, and motor development at one year corrected-age.

Furthermore, stepwise regression analyses were performed for those quantitative MR measures that revealed significant correlations with outcome scores. In order to account for potentially confounding effects of neonatal mortality risk and illness severity, statistical corrections were applied using a weighted logistic regression-based approach for the *Clinical Risk Index for Babies II score* (CRIB II) [25]. The CRIB II is a tool for the prediction of mortality risk and illness severity, which takes the following aspects into account: birth weight; GA at birth; body temperature; base excess; and sex [25]. Due to the small sample, this approach was chosen in order to allow weighting for multiple confounders summarized via a single controlling metric.

Due to the small sample size and the increased risk of a type II error, corrections for multiple testing were waived [26]. The results presented are based on the quantifications performed by observer 1. The results based on the quantifications performed by observer 2 are presented in the Supplementary Material (Supplementary Document).

## Results

Thirty-three/41 (80.5%) extremely preterm infants (female/male: 16/17; mean GA at birth (weeks + days): 26 + 1 (±0 + 3); mean post-menstrual age at MRI (weeks + days): 37 + 2 (±1 + 3)) were enrolled in this investigation (Table 1). Eight/41 (19.5%) extremely preterm infants were excluded from this study due to lack of follow-up data. All data were evaluated with regard to image quality by one neuroradiologist, with four years of experience with neonatal MRI. In all included data, image quality was perceived to be sufficient for further analysis.

### ICC Analysis

For T1R, the ICC results revealed excellent agreement between both observers (range: 0.926 (CI: 0.851–0.963) to 0.971 (CI: 0.932–0.986)).

For T2R, the ICC results ranged from good to excellent agreement between both observers (range: 0.859 (CI: 0.385–0.950) to 0.948 (CI: 0.894–0.974)).

For ADC, the ICC results ranged from moderate to excellent agreement between both observers (range: 0.662 (CI: 0.228–0.844) to 0.906 (CI: 0.777–0.957)).

For FA, the ICC results ranged from good to excellent agreement between both observers (range: 0.785 (CI: 0.084–0.925) to 0.990 (CI: 0.980–0.995)) (Supplementary Table 2).

### Pearson’s Correlation Analysis

For cognitive outcome, *r* ranged between 0.261 (*p* = 0.142) and 0.317 (*p* = 0.072) (T1R); 0.032 (*p* = 0.859) and 0.412 (*p* = 0.017) (T2R); −0.401 (*p* = 0.021) and 0.239 (*p* = 0.180) (ADC); and −0.200 (*p* = 0.263) and 0.134 (*p* = 0.456) (FA).

Significant correlations were observed between cognitive outcome scores collected at one year of age and T2R (*r* = 0.412; *p* = 0.017) and ADC (*r* = −0.401; *p* = 0.021) of the medulla oblongata, determined at term-equivalent ages.

For language outcome, *r* ranged between 0.036 (*p* = 0.842) and 0.128 (*p* = 0.477) (T1R); 0.185 (*p* = 0.303) and 0.333 (*p* = 0.058) (T2R); −0.121 (*p* = 0.504) and 0.148 (*p* = 0.412) (ADC); and −0.256 (*p* = 0.151) and 0.003 (*p* = 0.986) (FA).

There were no significant correlations between language outcome scores collected at one year of age and quantitative MR metrics, determined at term-equivalent ages.

For motor outcome, *r* ranged between 0.253 (*p* = 0.155) and 0.513 (*p* = 0.002) (T1R); 0.116 (*p* = 0.521) and 0.405 (*p* = 0.019) (T2R); −0.414 (*p* = 0.017) and 0.111 (*p* = 0.537) (ADC); and −0.352 (*p* = 0.045) and 0.062 (*p* = 0.730) (FA).

**Fig. 2:**
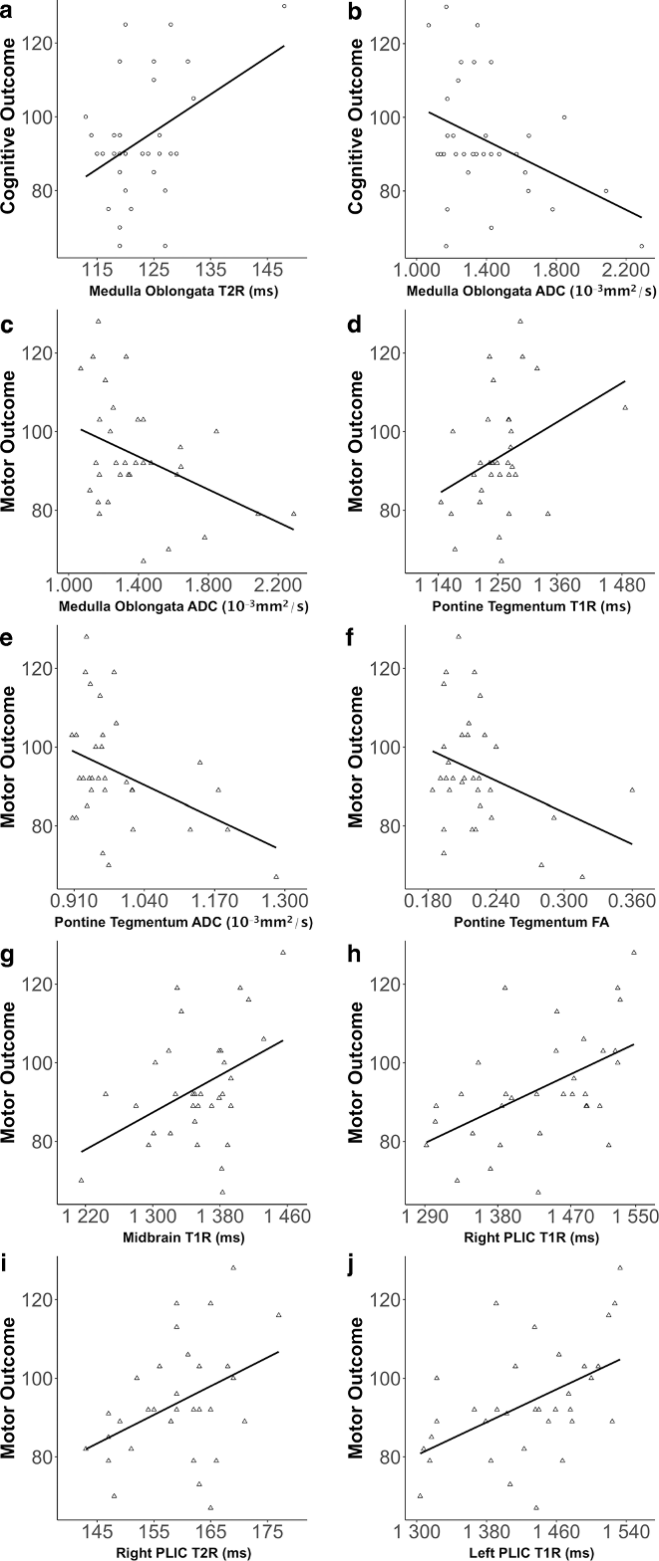
Demonstration of significant relationships between quantitative metrics (T1R (ms), T2R (ms), ADC (10^−3^ mm^2^/s), and FA) (x-axis) determined at term-equivalent ages by rater 1 and cognitive outcome data (**a**,**b**) and motor outcome data (**c**–**j**) (y-axis) collected at one year of age

Significant correlations were observed between motor outcome scores collected at one year of age and ADC (*r* = −0.408; *p* = 0.018) of the medulla oblongata; T1R (*r* = 0.346; *p* = 0.049), ADC (*r* = −0.414; *p* = 0.017), and FA (*r* = −0.352; *p* = 0.045) of the pontine tegmentum; T1R (*r* = 0.415; *p* = 0.016) of the midbrain; T1R (*r* = 0.513; *p* = 0.002) and T2R (*r* = 0.405; *p* = 0.019) of the right PLIC; and T1R (*r* = 0.504; *p* = 0.003) of the left PLIC, determined at term-equivalent ages (Fig. 2 and Supplementary Table 3).

### Stepwise Regression Analysis

To identify the most predictive imaging metrics for neurodevelopmental outcomes, only quantitative measures that revealed significant correlations with outcome data were considered for regression analysis.

For cognitive outcome, T2R and ADC (medulla oblongata) were included in the analysis. The model identified the ADC as the strongest predictor for cognitive development (model 1: R^2^ = 0.184; *p* = 0.013). However, the prognostic potential increased when the T2R was considered as a complementary variable (model 2: R^2^ = 0.296; *p* = 0.037).

**Fig. 3:**
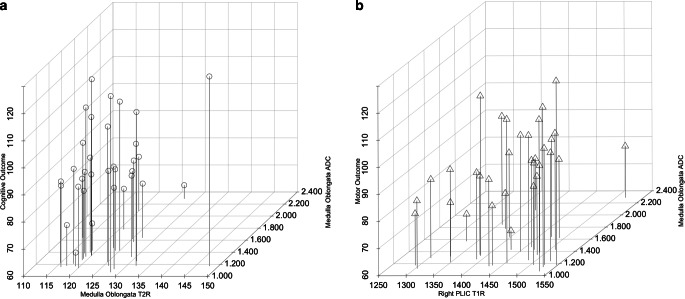
Illustration of the associations between quantitative metrics (determined by MRI near term-equivalent ages) (x-axes)—characterized by significant predictive potential for future neurologic development—and cognitive (**a**) and motor outcomes (**b**) (y-axis) assessed at one year of age

For motor outcome, T1R (pontine tegmentum, midbrain, right PLIC, and left PLIC), T2R (right PLIC), ADC (medulla oblongata and pontine tegmentum), and FA (pontine tegmentum) were included in the analysis. The model identified the T1R (right PLIC) as the strongest predictor for motor development (model 1: R^2^ = 0.251; *p* = 0.003). However, the prognostic potential increased when the ADC (medulla oblongata) was considered as a complementary variable (model 2: R^2^ = 0.405; *p* = 0.009) (Fig. 3).

## Discussion

In this study, the potential value of relaxometry- and DTI-based MR measures for neurologic outcome prediction was investigated in a cohort of extremely preterm infants. Interestingly, several imaging metrics obtained at term-equivalent ages showed strong relationships with cognitive and motor outcomes at one year corrected-age. Furthermore, stepwise regression analyses disclosed significant predictive potential for both relaxometry- and DTI-derived properties. Therefore, the findings presented here suggest that quantitative MRI at term-equivalent age may supply valuable data to the radiological assessment for the estimation of neurodevelopmental outcomes in extremely premature infants.

Relaxation times and diffusion tensor-based metrics are considered to change throughout the course of brain maturation [11, 14, 21]. Primarily, myelination processes appear to alter T1R/T2R and ADC/FA values over the fetal period and the first months of life [10, 21, 27–31]. Therefore, quantifications were performed in areas thought to reveal advanced patterns of brain myelination at early developmental stages [19, 20].

While the T1R is already decreased at the stage of “pre-myelination” (i.e., interactions between H_2_O and myelin building blocks (glycolipids and cholesterol) at the beginning of myelinogenesis), the T2R does not show considerable shortening until tightening of fully developed myelin sheaths occurs [27–29]. Thus, lower relaxation time metrics indicate advanced stages of myelination [10, 14]. However, the results of this investigation revealed correlations between relaxometry-based measures and neurologic outcomes, with higher T1R/T2R values at term-equivalent ages associated with better cognitive and motor development. Several studies demonstrated altered neuronal development in preterm infants, most likely due to adverse environmental effects on the fragile preterm brain postnatally [32–35]. There is evidence that preterm-delivery leads to accelerations in white matter maturation and increases in regional brain volumes, which underpins the theories of over-abundant connectivity and associated myelinogenesis in these patients [33, 36, 37]. Conversely, these expedited myelination processes could negatively affect further neurologic development—potentially due to accompanied alterations in neural plasticity—and, therefore, explain the observed positive relationships [2, 5, 32, 33]. In a previous study, similar correlations were observed between T1R determined in the frontal white matter at term-equivalent ages and cognitive outcomes collected 18 months after birth in a sample of former preterm neonates [33].

As brain myelination proceeds, diffusion tensor-derived metrics change due to increases in membrane density and ongoing myelin ensheathment [30, 38]. The ADC is related to the *Brownian* motion of H_2_O molecules [30, 38, 39]. Thus, due to restricted movement of water protons along myelinated fibers, the diffusivity decreases throughout the course of myelinogenesis [30, 31, 38]. In contrast, the FA measures the anisotropy of water molecules, which increases progressively as fiber ensheathment occurs [30, 38]. Therefore, advanced stages of myelination are associated with lower ADC and higher FA values [21, 29–31, 38]. In this study, significant relationships were observed between diffusivity measures and developmental scores, with lower ADC values associated with better cognitive and motor outcomes, which is in line with a previous investigation [40]. As demonstrated by Navarra et al., negative correlations were detected between diffusivity metrics and neurodevelopmental outcomes in former preterm infants [40]. These observations support the hypothesis that advanced maturational stages at term-equivalent ages correlate with a more favorable neurodevelopment. However, on the contrary, FA parameters revealed negative correlations with motor outcome data, which, particularly when taking into account the positive associations between relaxometry-based metrics and developmental scores, contradicts the aforementioned assertions. A potential explanation for the observed inconsistencies between relaxometric- and FA-based versus ADC-derived results may be due to the fact that the ADC is also considered to decrease due to increases of membrane density throughout development [21]. These changes of membrane structure do not only apply for myelin, but may also concern the axolemma, which covers the axon of the neurons. Therefore, the decreases in ADC values may be not regarded as a direct function of ongoing myelination and could be based on the proceeding alterations of general membrane structure within the course of development [21]. Nonetheless, although DTI- and relaxometry-based MRI appear to support rather different hypotheses in terms of developmental states at term-equivalent ages and favorable future outcomes, both modalities revealed promising predictive potential in this regard.

ADC metrics determined in the medulla oblongata were identified as potential predictors for cognitive and motor outcome scores. Furthermore, T1R (right PLIC) (motor outcomes) and T2R measures (medulla oblongata) (cognitive outcomes) revealed significant prognostic value. Since myelination proceeds caudally to rostrally, the medulla oblongata is considered to hold relatively huge quantities of mature fibers perinatally, which could explain the predictive potential of this region [19, 20]. Even though the medulla oblongata is not considered a brain area directly linked to cognition, the provided imaging metrics may serve as surrogate parameters for cognitive development [41]. Moreover, based on the quantifications performed by observer 2 (Supplementary Document), also the T2R of the right PLIC appears to be a surrogate marker for future cognitive performance. In contrast, several pathways of motor control take their course through the brainstem, which may be related to the prognostic value of diffusivity measures for motor outcomes [19, 20]. Furthermore, T1R of the right PLIC revealed significant results for motor outcome prediction, possibly explained by relations to corticospinal fibers that travel through this region [19, 20]. However, myelination of the pyramidal system progresses slowly, with mature myelin still scanty at term-equivalent ages [19, 20]. Nonetheless, T1R and ADC metrics are regarded as sensitive measures even at “pre-myelination” states [27–31]. The data determination performed by rater 2 (Supplementary Document) revealed consistent results with prognostic values for quantitative metrics obtained in motor-related regions (brainstem and PLIC). However, as opposed to cognitive and motor outcomes, no prognostic MR parameters for future language abilities were disclosed, most likely due to the relative immaturity of verbal functions at the early toddler stage [42]. Nevertheless, quantitative imaging measures with which to predict language development in infants with a preterm history are greatly needed and deserve further consideration.

The steadily decreasing mortality risk of preterm infants confront modern healthcare with hitherto unprecedented challenges [1, 4]. In particular, the potentially adversely affected neurologic development in these patients requires more attention from attending physicians [2–5]. The investigated quantitative MR approach may help to predict future outcomes in extremely preterm infants and, therefore, provides a modality with which to identify patients at risk for future neurologic deficits and to selectively establish appropriate treatments at the earliest stages of postnatal development. Thus, quantitative imaging supplies valuable information to the clinical decision-making, which may contribute to more favorable outcomes in premature infants. However, the optimization of postnatal and interdisciplinary management in these patients will remain challenging.

Several limitations require consideration. The sample size was small, without the availability of outcome data beyond one year corrected-age and a control cohort of term born infants that underwent the same procedures. Nonetheless, although some studies report low validity of outcome assessments at 12 months corrected-age [43], there is evidence that the appraisals of future neurologic performance at one year provide comparable results to those obtained at more advanced developmental stages [44]. While 3 Tesla MR systems are coming to the fore, even in pediatric imaging settings, the presented data are based on a 1.5 Tesla machine. This study focused on only a limited number of early-myelinating brain areas and quantitative imaging metrics [19, 20]. However, associations between quantitative metrics determined in “pre-myelinating” regions and neurodevelopmental outcomes were beyond the scope of this work. Moreover, within the framework of this investigation, it was aimed to focus only on clinically well-established MR parameters for myelin imaging (i.e., T1R, T2R, ADC, and FA) [21]. Furthermore, due to the retrospective nature of this study, the technical features of both quantitative sequences differed and appropriate co-registration solutions were not available. Thus, ROI placement was potentially performed on different slices. Therefore, multiple measures were performed for each brain section of interest to maintain the overlap for DTI- and relaxometry-derived metrics at its maximum across the different sequences [21]. Beyond that, technical optimizations are needed (e.g. isovoxel-based imaging, thinner slices, etc.) to make MDME-based MRI a complete stand-alone tool for modern pediatric MRI. However, further research on 3D-based relaxometric MRI is underway to overcome this profound limitation [45]. Although only infants in whom MRI was perceived to be without brain pathology were included, several conditions encountered within the investigated cohort (Table 1) may have affected brain development. Therefore, a statistical approach was applied that takes mortality risk and illness severity (CRIB II) into account, which enabled to statistically control for a variety of potential confounders via a single, substantial metric [25]. However, since various conditions associated with prematurity may impact myelin development, not all possible effects of causality were captured, since the rather small sample and the exploratory design prohibited the application of more conservative statistical approaches [26, 46, 47]. Finally, this study does not provide information on GA-related differences regarding neurodevelopmental outcomes within the group of extremely preterm newborns. Nonetheless, given the fact that the survival rates following extreme prematurity are rising steadily, this topic requires further consideration in the future [48].

In summary, the findings of this study indicate strong relationships between quantitative metrics determined by MRI near term and neurodevelopmental outcomes at one year corrected-age in children with a history of extreme preterm birth. Moreover, DTI and relaxometry-based imaging bear prognostic potential for the prediction of cognitive and motor outcomes in these fragile patients. Therefore, quantitative MRI represents a promising approach with which to estimate neurologic development, which may increase the value of routine brain imaging at term-equivalent ages in extremely preterm infants.

### Supplementary Information

**Supplementary Document: **Results based on the quantifications performed by observer 2

**Supplementary Table 1: **Neonatal MR protocol (< 30 min)

**Supplementary Table 2: **Inter-rater reliability (intra-class correlation coefficient)

**Supplementary Table 3: **Correlational analysis

## Abbreviations

FA: Fractional anisotropy
GA: Gestational age
ICC: Intra-class correlation coefficient
MDME: Multi-dynamic multi-echo
PLIC: Posterior limb of the internal capsule
ROI: Region of interest
T1R: T1-relaxation time
T2R: T2-relaxation time
