# Supplementary material for: Quantitative Magnetic Resonance Imaging for Neurodevelopmental Outcome Prediction in Neonates Born Extremely Premature—An Exploratory Study
Source: Clin Neuroradiol. 2024 Jan 30;34(2):421–9. doi: 10.1007/s00062-023-01378-9 (PMC11129968; doi:10.1007/s00062-023-01378-9)
Supplement: Supplementary file 1 — Supplementary Document: Results based on the quantifications performed by observer 2 [file 62_2023_1378_MOESM1_ESM.docx]

**Supplementary Document:** Results based on the quantifications performed by observer 2

*Pearson’s Correlation Analysis*

There were significant correlations between cognitive scores and *a)* T1R of the left PLIC [*r*=0.352 (*p*=0.045)] and *b)* T2R of the right PLIC [*r*=0.406 (*p*=0.019)].

There were no significant correlations between language scores and quantitative metrics.

There were significant correlations between motor scores and *a)* T1R of the midbrain [*r*=0.446 (*p*=0.009)], *b)* T1R [*r*=0.456 (*p*=0.008)] and T2R of the right PLIC [*r*=0.385 (*p*=0.027)], *c)* T1R of the left PLIC [*r*=0.477 (*p*=0.005)], and *d)* ADC of the pontine tegmentum [*r*=-0.348 (*p*=0.047)].

*Stepwise Regression Analysis*

The model identified the T2R of the right PLIC as the strongest predictor for cognitive development (R^2^=0.138; *p*=0.033).

The model identified the T1R of the left PLIC as the strongest predictor for motor development (model 1: R^2^=0.212; *p*=0.007). However, the prognostic potential increased when the ADC of the pontine tegmentum was considered as a complementary variable (model 2: R^2^=0.347; *p*=0.019).
