# Supplementary material for: Quantitative Magnetic Resonance Imaging for Neurodevelopmental Outcome Prediction in Neonates Born Extremely Premature—An Exploratory Study
Source: Clin Neuroradiol. 2024 Jan 30;34(2):421–9. doi: 10.1007/s00062-023-01378-9 (PMC11129968; doi:10.1007/s00062-023-01378-9)
Supplement: Supplementary file 2 — Supplementary Table 1: Neonatal MR protocol (< 30 min) [file 62_2023_1378_MOESM2_ESM.docx]

**Supplementary Table 1:** Neonatal MR protocol (<30 minutes)

| **Sequence** | **Plane** | **AT (min)** | **TR (ms)** | **TE (ms)** | **Voxel Size (mm)** | **FOV (mm)** | **Matrix (slices)** |
| --- | --- | --- | --- | --- | --- | --- | --- |
| T1 (3D) | Sag. | 03:46 | 25 | 7.6 | 0.75x0.75x2.00 | 120x120x99 | 160x160x99 |
| T1 SE (2D) | Ax. | 03:07 | 400 | 15 | 0.83x1.05x3.00 | 120x120x90 | 144x115x30 |
| T2 TSE (2D) | Ax. | 01:48 | 3000 | 140 | 0.94x1.06x3.00 | 120x120x102 | 128x113x34 |
| T2 TSE (2D) | Cor. | 01:48 | 3000 | 140 | 0.94x1.06x3.00 | 110x110x108 | 116x103x36 |
| T2 TSE (2D) | Sag. | 01:48 | 3000 | 140 | 0.94x1.06x3.00 | 120x120x108 | 128x113x36 |
| DWI (2D) | Ax. | 01:34 | 4066 | 90 | 1.14x1.15x3.00 | 200x200x92 | 176x170x28 |
| SWI (2D) | Ax. | 03:35 | 51 | 12 | 0.85x1.00x2.00 | 170x139x90 | 200x138x90 |
| MDME (2D) | Ax. | 05:24 | 3309 | 13; 100 | 0.89x1.04x4.00 | 200x165x109 | 224x159x22 |
| DTI (2D) | Ax. | 05:26 | 2435 | 88 | 2.41x2.48x2.50 | 164x164x100 | 68x66x40 |

AT: Acquisition time

DTI: Diffusion-tensor imaging

DWI: Diffusion-weighted imaging

FOV: Field-of-view

MDME: Multi-dynamic multi-echo

SE: Spin echo

SWI: Susceptibility-weighted imaging

TE: Echo time

TR: Repetition time

TSE: Turbo spin echo
