# Supplementary material for: Quantitative Magnetic Resonance Imaging for Neurodevelopmental Outcome Prediction in Neonates Born Extremely Premature—An Exploratory Study
Source: Clin Neuroradiol. 2024 Jan 30;34(2):421–9. doi: 10.1007/s00062-023-01378-9 (PMC11129968; doi:10.1007/s00062-023-01378-9)
Supplement: Supplementary file 3 — Supplementary Table 2: Inter-rater reliability (intra-class correlation coefficient) [file 62_2023_1378_MOESM3_ESM.docx]

**Supplementary Table 2:** Inter-rater reliability (intra-class correlation coefficient)

| **ROI** | **T1R** | **T2R** | **ADC** | **FA** |
| --- | --- | --- | --- | --- |
| **Left PLIC** | 0.926 (0.851–0.963) | 0.875 (0.745–0.938) | 0.662 (0.228–0.844) | 0.785 (0.084–0.925) |
| **Right PLIC** | 0.946 (0.828–0.978) | 0.948 (0.894–0.974) | 0.778 (0.551–0.890) | 0.821 (0.635–0.912) |
| **Midbrain** | 0.960 (0.906–0.981) | 0.914 (0.726–0.965) | 0.906 (0.777–0.957) | 0.968 (0.923–0.986) |
| **Pontine Tegmentum** | 0.971 (0.932–0.986) | 0.937 (0.860–0.970) | 0.680 (0.361–0.841) | 0.958 (0.884–0.982) |
| **Medulla Oblongata** | 0.943 (0.809–0.977) | 0.859 (0.385–0.950) | 0.759 (0.255–0.903) | 0.990 (0.980–0.995) |

Numbers in parentheses: 95% confidence intervals

ADC: Apparent diffusion coefficient

FA: Fractional anisotropy

PLIC: Posterior limb of the internal capsule

ROI: Region of interest

T1R: T1-relaxation time

T2R: T2-relaxation time
