# Supplementary material for: Quantitative Magnetic Resonance Imaging for Neurodevelopmental Outcome Prediction in Neonates Born Extremely Premature—An Exploratory Study
Source: Clin Neuroradiol. 2024 Jan 30;34(2):421–9. doi: 10.1007/s00062-023-01378-9 (PMC11129968; doi:10.1007/s00062-023-01378-9)
Supplement: Supplementary file 4 — Supplementary Table 3: Correlational analysis [file 62_2023_1378_MOESM4_ESM.docx]

**Supplementary Table 3:** Correlational analysis

| **ROI** | **Cognitive Scores^a^** | **Language Scores^a^** | **Motor Scores^a^** |
| --- | --- | --- | --- |
| **Left PLIC** |  |  |  |
| T1R^b^ | *r*=0.302 (*p*=0.087) | *r*=0.128 (*p*=0.477) | *r*=0.504 (*p*=0.003) |
| T2R^b^ | *r*=0.063 (*p*=0.726) | *r*=0.242 (*p*=0.175) | *r*=0.167 (*p*=0.353) |
| ADC^b^ | *r*=0.086 (*p*=0.634) | *r*=0.045 (*p*=0.805) | *r*=0.014 (*p*=0.937) |
| FA^b^ | *r*=-0.057 (*p*=0.752) | *r*=-0.256 (*p*=0.151) | *r*=0.062 (*p*=0.730) |
| **Right PLIC** |  |  |  |
| T1R^b^ | *r*=0.313 (*p*=0.076) | *r*=0.053 (*p*=0.768) | *r*=0.513 (*p*=0.002) |
| T2R^b^ | *r*=0.335 (*p*=0.057) | *r*=0.292 (*p*=0.099) | *r*=0.405 (*p*=0.019) |
| ADC^b^ | *r*=0.239 (*p*=0.180) | *r*=-0.121 (*p*=0.504) | *r*=0.111 (*p*=0.537) |
| FA^b^ | *r*=-0.200 (*p*=0.263) | *r*=-0.202 (*p*=0.260) | *r*=0.020 (*p*=0.914) |
| **Midbrain** |  |  |  |
| T1R^b^ | *r*=0.317 (*p*=0.072) | *r*=0.036 (*p*=0.842) | *r*=0.415 (*p*=0.016) |
| T2R^b^ | *r*=0.154 (*p*=0.394) | *r*=0.216 (*p*=0.227) | *r*=0.116 (*p*=0.521) |
| ADC^b^ | *r*=-0.332 (*p*=0.059) | *r*=0.148 (*p*=0.412) | *r*=-0.325 (*p*=0.065) |
| FA^b^ | *r*=0.134 (*p*=0.456) | *r*=-0.089 (*p*=0.621) | *r*=-0.046 (*p*=0.799) |
| **Pontine Tegmentum** |  |  |  |
| T1R^b^ | *r*=0.308 (*p*=0.081) | *r*=0.069 (*p*=0.704) | *r*=0.346 (*p*=0.049) |
| T2R^b^ | *r*=0.032 (*p*=0.859) | *r*=0.333 (*p*=0.058) | *r*=0.155 (*p*=0.389) |
| ADC^b^ | *r*=-0.229 (*p*=0.200) | *r*=0.000 (*p*=0.998) | *r*=-0.414 (*p*=0.017) |
| FA^b^ | *r*=0.003 (*p*=0.986) | *r*=-0.098 (*p*=0.586) | *r*=-0.352 (*p*=0.045) |
| **Medulla Oblongata** |  |  |  |
| T1R^b^ | *r*=0.261 (*p*=0.142) | *r*=0.037 (*p*=0.836) | *r*=0.253 (*p*=0.155) |
| T2R^b^ | *r*=0.412 (*p*=0.017) | *r*=0.185 (*p*=0.303) | *r*=0.338 (*p*=0.054) |
| ADC^b^ | *r*=-0.401 (*p*=0.021) | *r*=-0.048 (*p*=0.790) | *r*=-0.408 (*p*=0.018) |
| FA^b^ | *r*=0.001 (*p*=0.996) | *r*=0.003 (*p*=0.986) | *r*=-0.184 (*p*=0.305) |

^a^ Outcome data determined at one year of age

^b^ Quantitative imaging data determined at term-equivalent ages

ADC: Apparent diffusion coefficient

FA: Fractional anisotropy

PLIC: Posterior limb of the internal capsule

ROI: Region of interest

T1R: T1-relaxation time

T2R: T2-relaxation time
